# The mechanism of translation

**DOI:** 10.12688/f1000research.9760.1

**Published:** 2017-03-01

**Authors:** Joachim Frank

**Affiliations:** 1 Department of Biochemistry and Molecular Biophysics, Columbia University, New York, NY, USA; 2 Department of Biological Sciences, Columbia University, New York, NY, USA; 3 Howard Hughes Medical Institute, Columbia University, New York, NY, USA

**Keywords:** cryo-EM, translation, smFRET, x-ray crystallography

## Abstract

Translation of the genetic code on the ribosome into protein is a process of extraordinary complexity, and understanding its mechanism has remained one of the major challenges even though x-ray structures have been available since 2000. In the past two decades, single-particle cryo-electron microscopy has contributed a major share of information on structure, binding modes, and conformational changes of the ribosome during its work cycle, but the contributions of this technique in the translation field have recently skyrocketed after the introduction of a new recording medium capable of detecting individual electrons. As many examples in the recent literature over the past three years show, the impact of this development on the advancement of knowledge in this field has been transformative and promises to be lasting.

## Introduction

In all organisms on earth, translation of the genetic code into protein is performed on the ribosome, a molecular machine of extraordinary complexity that is composed of RNA and a large (∼80) number of proteins. Animals have them, plants have them, bacteria have them—all in different versions but with a common, virtually identical core. As a restless machine essential to life, it brings to mind our heart, the restlessness we experience every moment we are awake.

Since its discovery and first visualization in the 1960s, as an electron-dense granule studding the membrane of the endoplasmic reticulum ^1^, the ribosome has been subject to many biochemical and biophysical studies that attempted to elucidate the mechanism of protein synthesis. Central to these studies were attempts at solving the ribosome structure. Once its structure was solved, so the thinking went, the way the ribosome reads the code and accordingly strings the amino acids up to form polypeptide—the precursor of the fully folded protein—would be revealed. The German language has a saying, adopted from the New Testament, “Wie Schuppen von den Augen fallen” (to fall like scales from the eyes), meaning that a long-sought solution to a problem becomes evident in a single flash of insight. But in the case of the ribosome, no such epiphany occurred for quite some time. Instead, hard work was required, drawing from x-ray crystallography, cryo-electron microscopy (cryo-EM), and mutation studies and increasingly by enlisting the help of other techniques capable of supplying information on the dynamics in real time, notably single-molecule fluorescence resonance energy transfer (smFRET).

As background for the current explosive development of structural and functional knowledge in the ribosome field, it is of interest to recall the relative contributions of electron microscopy and x-ray crystallography to the elucidation of ribosome structure. Electron microscopy employing negative staining provided first visualizations, sufficient to show the two subunits with different sizes. Attempts by several groups to obtain well-ordered two-dimensional crystals, suitable for electron crystallography, were only moderately successful ^2, 3^. Meanwhile, x-ray crystallography made very slow progress because of the difficulties posed by the crystallographic phasing of both the 30S and 50S subunits, requiring new approaches of labeling with compounds of sufficient phasing power. The first success in visualizing the subunits, their connecting bridges, and the intricate topology of the intersubunit space came with the single-particle cryo-EM approach ^4, 5^. Subsequently, initial structures relevant to the functional mechanism, showing the ribosome engaged with tRNAs and elongation factors, started to appear ^6– 9^. In the year 2000, subsequently referred to as the *annus mirabilis* of ribosome research, three x-ray structures appeared: one of the large subunit of an archaea bacterium ^10^, the other two of the small subunit of a thermophilic bacterium ^11, 12^. These studies provided the first atomic models, revealing daunting complexity. However, critical information was missing not only for understanding the mechanism of action but even for a satisfactory characterization of the functional sites.

## The translation field up to 2013

To understand the mechanism of translation on an elementary level, one has to figure out the structural basis for three events that are repeated for every single *codon* (that is, the element of the genetic code residing on the mRNA): (i) decoding, or the recognition of the current codon with the help of a cognate tRNA; (ii) peptidyl transfer, or the way the new-coming amino acid is being linked to the nascent polypeptide; and (iii) mRNA-tRNA translocation, or the way the ribosome manages to move on to the next codon.

Ostensibly, the solution of each of these problems required, at the very least, the atomic structures of the *complete* ribosome bound with various combinations of tRNAs and elongation factors in a functional context. For instance, conclusions on the mechanism of peptidyl-transfer made on the basis of the large subunit with substrates bound ^13^ did not stand up to subsequent scrutiny (for example, 14). Such structures, however, were not available for quite some time. The first x-ray structure of the complete ribosome came out in 2001 ^15^ and contained three bound tRNAs, but its resolution was relatively low (5.5 Å), requiring the subunit structures published the year before for interpretation and atomic model building. Another structure from the same group provided the first mapping of the path of mRNA ^16^. Then, for a number of years, a frustrating situation prevailed where functionally meaningful information was provided mainly by single-particle cryo-EM with increasingly better resolution but falling short, by a considerable margin, of the resolution required to pinpoint the crucial interactions between the various molecular players. X-ray crystallography, on the other hand, provided a few important structures in which mRNA and tRNAs were fortuitously bound to a model mRNA (for example, 17, 18), establishing tRNA-ribosome interactions in the pre-translocational state and pinpointing the role of a magnesium ion in stabilizing a kink in the mRNA at the decoding center ^17^.

To make use of knowledge provided by both techniques of structure determination, the hope was set on so-called hybrid methods ^19^ (that is, by interpreting cryo-EM density maps in terms of available, suitably modified x-ray models ^20– 23^). An example of radical change in structure from the free form studied by x-ray crystallography to its ribosome-bound structure visualized by cryo-EM was provided by release factors RF1 ^24^ and RF2 ^20^, illustrating the need for methods of flexible fitting.

Of course, the ribosome was only one of many structures facing the same quandary (that is, low-resolution density maps of functional states versus high-resolution atomic models of components). Starting at the turn of the century, “hybrid” conferences (for example, “The Structure of Large Biological Complexes” ^25, 26^) were organized with the aim of bringing the communities of structural biologists together with those specialized in modeling, signal processing, and molecular dynamics simulations. Overall, these meetings were highly successful in creating an awareness of the complexity of the overall goal and in seeking solutions by connecting and integrating the methods employed in different fields.

In regard to tRNA selection and decoding, the basic principle by which the ribosome ascertains the formation of Watson-Crick pairing in the cognate case was settled early on ^27, 28^ and confirmed by many observations made since. The way the tRNA enters the ribosome as part of the ternary complex with elongation factor Tu (EF-Tu) and GTP was first observed by cryo-EM, with the surprising conclusion that incoming tRNA is strongly deformed, constituting a molecular spring ^29– 31^, suggesting that its latent energy sets the threshold for cognate versus near-cognate codon selection ^32^. Evidence that this mechanism is at work for the different classes of tRNA was later supplied by Li *et al.* ^33^. Such structures showing the tRNA in the so-called A/T position were captured at the atomic level by x-ray crystallography using kirromycin or non-hydrolyzable GTP analogs ^14, 34^, supplying significant insight into the way GTP hydrolysis is triggered following recognition of a cognate codon at the decoding center. At that time, the best resolution achieved by single-particle cryo-EM of the A/T complex was in the range of 6 to 7 Å ^35^, requiring flexible fitting of known structures for interpretation ^22^. Insights into the stochastic nature of tRNA selection in real time were provided in pioneering work by the Puglisi group ^36, 37^.

Translocation of mRNA-tRNA is a multi-step process of high complexity, during which the moiety formed by mRNA and two tRNAs bound to it via codon-anticodon interaction is moved along by the precise distance of one codon. Over a period of more than a decade, many structures shedding light on this process were obtained by cryo-EM and x-ray crystallography, starting with the observation of a ratchet-like intersubunit motion ^38, 39^ in apparent response to elongation factor G (EF-G; eEF2 in eukaryotes) binding, and bringing increasing evidence for the existence of intermediate states ^40– 44^. Real-time recordings of FRET signals from individual ribosomes with strategically placed donor/acceptor combinations ^45^ reported on the motions of the molecular machine and the way it is affected by binding of EF-G. Altogether, the state of knowledge three years ago on the events of the elongation cycle has been portrayed in an extensive review by Voorhees and Ramakrishnan ^46^.

## The effect of the “resolution revolution” in the field of translation

As we have seen, single-particle cryo-EM even before the advent of the direct electron detectors has vastly expanded the scope and potential for the elucidation of the mechanism of translation. With this technique, many discoveries of functional states and conformational dynamics have been made since the turn of this century; however, as a result of new electron recording technology, the past three years in particular have brought an explosion of new information at resolutions in the range of 2.5 to 4 Å—resolutions that in principle permit the building of atomic models without resorting to published x-ray structures (but see the caveat below). In several ways, this breakthrough development has simplified the interpretation of density maps since reliance on hybrid methods has become less important. On the other hand, the increase in resolution and the sharpening of tools for sorting and classification of heterogeneous data ^47– 49^ have meant that for each project a plethora of structures in different states—not just a single one—are created, presenting new challenges of interpretation.

This situation is in some ways reminiscent of a phenomenon in high-energy physics, which has seen a proliferation of different types of particles with ever more exotic qualities over the past two decades as the energy of beams in the colliders was raised. In a similar way, the number of observed states of the ribosome is steadily increasing as the resolution of three-dimensional visualization improves, posing new questions at each turn.

A look at the literature in the past three years indicates that the effect of these new technological developments in electron microscopy on the exploration of the translation mechanism is transformative, multi-fold, and still emerging. The ribosome structure itself, at resolutions better than 3 Å, readily reveals features never seen before by cryo-EM: inventories of rRNA modification sites, locations of Mg ^2+^ ions, and even water molecules ^50– 53^. It follows that fundamental multi-step mechanisms at the three functional centers can now be studied in much more detail than before. Again, it must be emphasized that owing to the capability of single-particle cryo-EM to capture molecules in their native states, high-resolution features such as locations of coordinating ions depicted are highly relevant for mechanistic interpretation of a molecular machine such as the ribosome.

**Figure 1.**
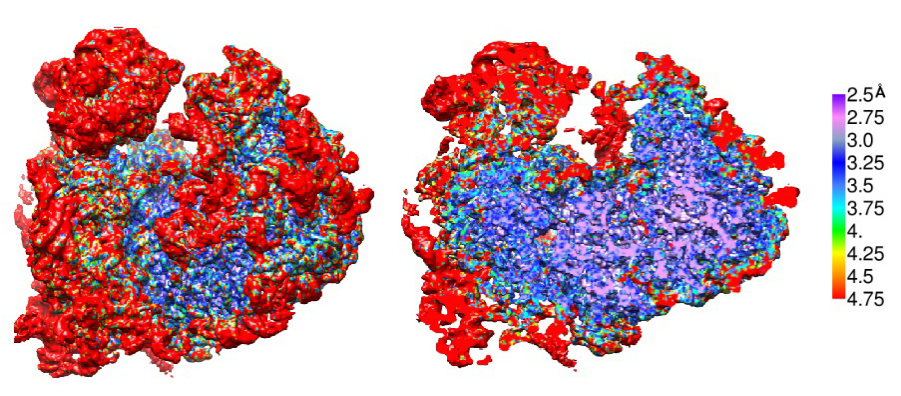
Cryo-electron microscopy map of the 80S ribosome from *Trypanosoma cruzi* at 3.2-Å resolution, before sharpening, colored by local resolution (see color key). (Left) Surface view. (Right) Central cut-away view. Like ribosomes from all Trypanosomatids, the *T. cruzi* ribosome possesses extra-large rRNA expansion segments, which all show up on the large subunit (on the right in each panel) as peripheral masses with high mobility. The density map shown (Liu *et al.*, unpublished) was obtained from 235,000 particle images (Liu *et al.* ^51^) after further refinement.

However, it must be noted here that the advantage presented by the ability to look at the free molecule outside the crystal context comes at a price: as a rule, peripheral regions have reduced resolution compared with the core. Therefore, *de novo* modeling, in cases where this has been attempted, usually starts in the core but often needs to stop before reaching the periphery, where modeling must rely on published x-ray structures which are not plagued by this limitation. An example is presented by our recent reconstruction of the 80S ribosome from *Trypanosoma cruzi* ^51^. Separate refinements of the 40S and 60S subunits yielded density maps at average resolutions of 3.7 and 2.5 Å, respectively, the difference in resolution reflecting high internal flexibility of the small subunit. The large subunit itself displayed resolutions ranging from 2.3 Å in the core to 4.6 Å at the periphery. Approximately 85% of the map was of sufficient quality for *ab initio* modeling. However, a significant saving of time was achieved by following a different strategy ^54^ making use of both the x-ray structure of the ribosome from yeast and a 5.5-Å cryo-EM structure of the ribosome from *T. brucei* and making suitable substitutions in non-conserved regions. A 3.2-Å reconstruction of the entire *T. cruzi* 80S ribosome obtained by further refinement ( Figure 1) demonstrates the large spread in local resolutions, ranging from 2.5 Å in the core of the large subunit to 4.75 Å in some of its rRNA expansion segments and in parts of the small subunit ( Figure 1).

With regard to mRNA-tRNA translocation, the challenge has been to explain the sequence of events as a result of the interplay between EF-G/eEF2 and the ribosome buffeted by Brownian motion. As to the initial binding of EF-G, an unexpected result was obtained by X-ray crystallography showing a compact conformation of the factor ^55^. Further work, both with x-ray crystallography ^40, 42– 44^ and high-resolution cryo-EM ^56, 57^, revealed intermediate states in translocation with fully engaged EF-G/eEF2. Novel insights were provided by both smFRET experiments, that reported on the interaction between the ribosome and EF-G ^58, 59^, or by using a two-wavelength method, simultaneously acting on both EF-G binding and the ribosome rotation state ^60^.

Because of a lack of x-ray structures, the understanding of the structural basis for IRES (internal ribosome entry site)-mediated translation has long had to rely on structures by cryo-EM at relatively low resolution, starting with the study by Spahn *et al.* of the hepatitis C virus IRES-bound 40S subunit ^61^. Knowledge in this area has been considerably advanced in four recent studies, two of which focused on cricket paralysis virus IRES ^62, 63^ and one on the IRES of hepatitis virus C ^64^. For the IRES element from Taura syndrome virus, inchworm-like translocation was observed in stunning detail by Abeyrathne *et al.* ^65^ in a series of six reconstructions, with resolutions in the range of 3.5 to 4.2 Å. What we have learned from these new results is the way the IRES RNA mimics mRNA and tRNA and interacts with the 80S ribosome to trigger conformational changes akin to those associated with regular mRNA-tRNA translocation in the host.

In the short time since the direct electron detectors came on the market, we have also seen an expansion of the scope of inquiry from model systems ( *Escherichia coli*, *Thermus thermophilus*, rabbit, and yeast) to ribosomes from a larger variety of species. These include a number of eukaryotic parasites ( *Trypanosoma cruzi* ^51^, *Leishmania* ^52, 53^, and *Plasmodium falciparum* ^66, 67^) as there is now reasonable hope that the emerging structures may be of help in the design of more effective drugs. Here, the advance in resolution is strikingly exemplified by comparison of the best density map achieved for *Trypanosoma brucei* (5.5 Å using conventional recording on film ^68^), with those for *T. cruzi* (2.5 Å ^51^ [ Figure 1]) and *Leishmania* (2.8 Å ^52^, 2.9 Å ^53^) using recording on direct electron detectors. In a similar vein, the first atomic structures of mitochondrial ribosomes have now been determined ^69– 72^, again spurring hope that diseases relating to dysfunctions of mitochondrial translation may become understood and treatable.

One of the most promising and exciting developments set in motion by the high-resolution breakthrough is the exploration of ribosome biogenesis, a vast field as hundreds of factors are involved in ribosome assembly and quality testing. Prior to the introduction of the new detectors, the first cryo-EM results showing assembly intermediates in 40S subunit biogenesis were obtained by the Karbstein lab ^73^. Examples of observations and discoveries made in recent high-resolution cryo-EM studies include the assembly pathway of Trypanosomatids ^51, 52^, large-scale domain motions during maturation of the 60S subunit ^74^ and evidence ^75, 76^ supporting the test drive paradigm of ribosome biogenesis ^77, 78^. Also worth mentioning in this context is a recent cryo-EM study which elucidates the structure of the earliest precursor of the eukaryotic ribosome, the 90S pre-ribosome ^79^.

In conclusion, the past three years have seen an extraordinary development of structural information relevant to the understanding of the mechanism of translation and translational regulation, fueled by the advent of the new detectors in electron microscopy. The fact that cryo-EM reconstructions can depict single molecules in a close-to-native environment and in a spectrum of multiple authentic states gives extra credence to structures derived from them. As a result of the increasing volume of depositions of relevant high-resolution cryo-EM density maps and coordinates derived from them in the public database, the overall pace of research and the potential for gaining new knowledge by interpreting and integrating this information have already picked up dramatically and will do so for some time to come.

## Abbreviations

cryo-EM, cryogenic electron microscopy; EF-G, elongation factor G; FRET, fluorescence resonance energy transfer; IRES, internal ribosome entry site; smFRET, single-molecule fluorescence resonance energy transfer.
